# Modelling the Effectiveness of Epidemic Control Measures in Preventing the Transmission of COVID-19 in Malaysia

**DOI:** 10.3390/ijerph17155509

**Published:** 2020-07-30

**Authors:** Balvinder Singh Gill, Vivek Jason Jayaraj, Sarbhan Singh, Sumarni Mohd Ghazali, Yoon Ling Cheong, Nuur Hafizah Md Iderus, Bala Murali Sundram, Tahir Bin Aris, Hishamshah Mohd Ibrahim, Boon Hao Hong, Jane Labadin

**Affiliations:** 1 Institute for Medical Research (IMR), Ministry of Health, Kuala Lumpur 50588, Malaysia; drbsgill@moh.gov.my (B.S.G.); lssarbhan@moh.gov.my (S.S.); sumarni.mg@moh.gov.my (S.M.G.); cheongyl@moh.gov.my (Y.L.C.); nuurhafizah@moh.gov.my (N.H.M.I.); bala.murali@moh.gov.my (B.M.S.); tahir.a@moh.gov.my (T.B.A.); 2 Department of Social and Preventive Medicine, Medical Faculty, University Malaya, Kuala Lumpur 50603, Malaysia; vivekjason1987@gmail.com; 3 Ministry of Health, Malaysia, Putrajaya 62590, Malaysia; drhishamshah@moh.gov.my; 4 Faculty of Computer Science and Information Technology, Universiti Malaysia Sarawak, Kota Samarahan 94300, Malaysia; wenhao0117@hotmail.com

**Keywords:** COVID-19, mathematical modeling, susceptible, exposed, infectious, and recovered (SEIR), isolation, movement

## Abstract

Malaysia is currently facing an outbreak of COVID-19. We aim to present the first study in Malaysia to report the reproduction numbers and develop a mathematical model forecasting COVID-19 transmission by including isolation, quarantine, and movement control measures. We utilized a susceptible, exposed, infectious, and recovered (SEIR) model by incorporating isolation, quarantine, and movement control order (MCO) taken in Malaysia. The simulations were fitted into the Malaysian COVID-19 active case numbers, allowing approximation of parameters consisting of probability of transmission per contact (*β*), average number of contacts per day per case (*ζ*), and proportion of close-contact traced per day (*q*). The effective reproduction number (R_t_) was also determined through this model. Our model calibration estimated that (*β*), (*ζ*), and (*q*) were 0.052, 25 persons, and 0.23, respectively. The (R_t_) was estimated to be 1.68. MCO measures reduce the peak number of active COVID-19 cases by 99.1% and reduce (*ζ*) from 25 (pre-MCO) to 7 (during MCO). The flattening of the epidemic curve was also observed with the implementation of these control measures. We conclude that isolation, quarantine, and MCO measures are essential to break the transmission of COVID-19 in Malaysia.

## 1. Introduction

COVID-19 is a novel pathogen first reported in Wuhan, Hubei Province, China, in December 2019. The World Health Organization (WHO) declared it as a global pandemic on 11 March 2020 [1] and the virus had infected more than 275,000 individuals and killed more than 11,000 people as of 22 March 2020 [2]. The cases and deaths are largely centered in China, South Korea, Italy, and Iran [2]. Despite intensive public health efforts in early detection, isolation, quarantine, treatment, contact tracing, and social-distancing measures in breaking the chain of transmission, there is a rise in COVID-19 cases and deaths worldwide [3].

Several studies have reported forecasts of the COVID-19 epidemic using various mathematical models both in China and globally [4,5,6,7,8,9]. A large number of studies utilized compartmental models, such as a susceptible, exposed, infectious, and recovered (SEIR) model, which is useful in the estimation of disease dynamics and forecasting of future cases [4,9,10,11,12,13,14,15]. SEIR models estimated in China, which are largely accurate, predicted a maximum of 227,989 cases by late February 2020, subsequently decreasing to 8042 cases by early March 2020 [4,9,11,15].

Despite being a novel virus [4], to date, several studies have already estimated epidemiological parameters for COVID-19 infections, which include the basic reproduction number (R0) and incubation period [11,16,17]. It is now evident that efficient human-to-human transmission of COVID-19 exists [18]. The WHO has determined an incubation period of 0–14 days for COVID-19, but it remains uncertain as to whether those without symptoms can spread the virus [16,18]. Earlier studies have reported incubation periods that fall within the range (0–14 days) estimated by the WHO [4,5,6,7,8,9], except for one recent study that estimates a longer incubation period of 25 days [19].

The R0 is a measure of transmissibility of an infection that estimates the number of cases on average for which an infected person will infect during their infectious period. More importantly, R0 > 1 indicates that the outbreak is self-sustaining unless effective control measures are implemented, while R0 < 1 indicates that the number of new cases decreases over time and eventually halts the outbreak [5]. The R0 estimated by the WHO for COVID-19 was 1.4–2.5, which was lower compared to the estimates reported during the Severe Acute Respiratory Syndrome (SARS) outbreak (R0 = 3.5) but higher than Middle East Respiratory Syndrome (MERS) (R0 = 0.8) outbreak; however, the case fatality rate for COVID-19 was much lower at 3% compared to SARS and MERS [3,20]. Other studies have reported higher R0 values ranging from 2.2–4.7 [4,5,6,7,8,9], which may have been an overestimate of the transmissibility of COVID-19 [18].

Currently, with no vaccine or effective treatment available, the only control measure for preventing disease transmission is by instituting several non-pharmaceutical interventions (NPIs). This includes movement control measures, early detection, contact tracing, isolation, quarantine, good hygiene practices, and use of personal protective equipment (PPE), which decreases the disease spread. Containment measures, such as quarantine of asymptomatic individuals and isolation of symptomatic individuals, are widely used for several infectious diseases, such as SARS [21], Ebola [22], and Measles.

The Malaysian health authorities have instituted several NPIs in order to lower the risk of transmissibility. Therefore, this paper aims to model the transmission dynamics of COVID-19 by incorporating the quarantine, isolation (instituted at the start of the outbreak), and movement control measures (implemented on 18 March 2020) currently undertaken in Malaysia. In addition, infectious disease modelling can further assist the health authorities in making informed public health policy decisions [23].

## 2. Materials and Methods

### 2.1. Data Source

The Malaysian case data for COVID-19 from 25 January to 17 March 2020 included active cases, duration of quarantine, and death rates, which were used in the estimation of parameters and were sourced from the Crisis Preparedness and Response Centre (CPRC), Ministry of Health, Malaysia. The remaining Malaysian active cases till 28 April 2020 were obtained from the press statements obtained from the Ministry of Health Malaysia (MOH) official website at http://www.moh.gov.my/. The sources of other parameters used in the model were obtained from previous studies.

### 2.2. Data Analysis

The model analysis provided outbreak simulation that included duration of outbreak, outbreak-peak in months, and peak active case numbers during the outbreak. These simulations were conducted when no control measures were taken and when movement control measures were taken. In addition, reduction in percentages of peak active cases with different control measures instituted were analyzed and described.

### 2.3. Model Formulation

Following a deterministic SEIR model, the exposed compartment plays an important role in contributing to the potential exponential transmission of COVID-19, as has been observed in China [4]. In doing so, we made five important assumptions [23]:

(1) We assumed that international travel had introduced transmission into the local setting but then played no further role in local transmissions, assuming a closed population. This was mitigated by travel restrictions and entry/exit point screening, which were enforced on 25 January 2020, limiting importation of cases.
(2) Malaysia’s total population, denoted as *N*, was divided into an extended SEIR compartmental model, distinguishing between the traced and untraced populations and incorporating the current control measures and precautions taken. Apart from the basic compartments of SEIR (Susceptible (*S*), Exposed (*E*), Infected (*I*), and Recovered (*R*)), this model had additional three compartments, namely traced close-contact and a negative test result population (*T*), traced exposed close-contact and positive test result population (*E_q_*), and the infected isolated (*I_q_*). It was assumed that, initially, the entire Malaysian population was susceptible, hence *S*_0_ = *N*.
(3) All Malaysian residents were assumed to be of equal measure in their likelihood to contract and transmit the virus, assuming there was homogenous mixing within the population; however, we assumed that only 67% of the population would be susceptible based on the concept of herd immunity [24]. Current literature on transmission of COVID-19 has suggested that there is no strong evidence to support asymptomatic transmission and therefore our model only accounted for symptomatic transmission [24].
(4) A constant population was assumed due to the short time period for the model development and projection, wherein changes of birth and death rates would be negligible.
(5) Some of the parameters used were developed based on the outbreak data in China. As such, we assumed homogeneity of the disease dynamics between China and Malaysia.

Based on the assumptions outlined above, the transmission model of COVID-19 that includes outbreak control measures in Malaysia was formulated using the extended SEIR model, which adopted parameters of control measures instituted during the SARS outbreak in Japan [24,25,26,27,28,29,30,31,32,33,34,35,36,37,38,39,40,41,42,43,44,45,46,47]. A recent paper has also introduced a model that encapsulates the non-pharmaceutical interventions in great detail [48]. The authors demonstrated that high compliance of using face masks may lead to the eradication of COVID-19. Similar to our model, we introduced two important parameters in the force of infection, which were the number of contacts made per patient per day, denoted as Zeta (ζ), and Kappa (*κ*), which was defined as the proportion of exposed people who take effective precautionary measures. Thus, the parameter ζ represents the effect of social distancing and the parameter κ reflects the proportion of the exposed people complying to practicing hand-hygiene, use of face masks, and any form of individual effective precautionary measures [24], which depicts the model in simulating the control measures taken in preventing COVID-19 transmission in Malaysia. Prior to the appearance of symptoms, a COVID-19 patient is mobile and free to interact with other susceptible persons. Individuals who have made contact with infectious individuals without taking effective precautionary measures will become infected with the probability denoted as Beta (*β*). This means that *β* signifies the probability of transmission per contact.

**Figure 1:**
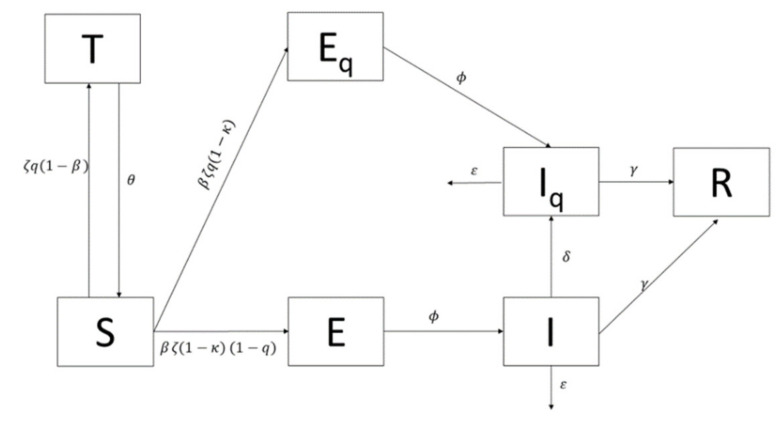
The extended susceptible, exposed, infectious, recovered model depicting the control measures taken in Malaysia. The additional compartments are the traced close-contact and a negative test result population (*T*), the traced exposed close-contact and positive test result population (*E_q_*) undergoing quarantine, and the infected isolated (*I_q_*).

The identification of the index case would trigger public health authorities to initiate contact tracing, with those identified persons being moved into the *E_q_* compartment if they are positive with or without symptoms. However, those identified persons who tested negative would be moved to the *T* compartment with a probability of transmissibility of 1 − *β*, and the rate of close contact traced per day was denoted as *q*. The close contact traced susceptible in *T* was released back to *S* after θ days. Therefore, the rate of tracing of those contacted with cases, but untraced by the public health, was 1 − *q* and was considered as exposed and thus moved to the *E* compartment and transferred to *I* after the incubation period (*φ*). The *I* population would be isolated and moved to Iq if they were detected with the proportion of the detection, which is denoted as *δ* and will not contribute further to the disease transmission [2]. Therefore, setting the parameters *q* and *δ* as zero (0) represents the fact that the no-tracing measure was taken and hence reverted back to the basic SEIR model. Case fatalities from the infection were removed from both the infected *I* and isolated *I_q_* compartments at a rate of *ε*. Infected individuals that survived were transferred into the *R* compartment at a rate of gamma (*γ*), which signifies the infectious rate. and hence the model assumes that immunity is attained after the infectious period. The differential equations (Equations (1)–(7)) that describe the dynamics of COVID-19 in human populations were formulated based on the compartmental diagram described in Figure 1. The description of the parameters and their corresponding values used in the model simulation are described in Table 1.

(1) $$\frac{dS}{dt}=\theta T-\frac{\beta \zeta q\left(1-\kappa \right)SI}{N}-\frac{\beta \zeta \left(1-q\right)\left(1-\kappa \right)SI}{N}-\frac{\zeta q\left(1-\beta \right)SI}{N},$$

(2) $$\frac{dT}{dt}=\frac{\zeta q\left(1-\beta \right)SI}{N}-\theta T,$$

(3) $$\frac{dE}{dt}=\frac{\beta \zeta \left(1-q\right)\left(1-\kappa \right)SI}{N}-\phi E,$$

(4) $$\frac{dI}{dt}=\phi E-\varepsilon I-\delta I-\gamma I,$$

(5) $$\frac{dE_{q}}{dt}=\frac{\beta \zeta q\left(1-\kappa \right)SI}{N}-\phi E_{q},$$

(6) $$\frac{dI_{q}}{dt}=\phi E_{q}+\delta I-\gamma I_{q}-\epsilon I_{q},$$

(7) $$\frac{dR}{dt}=\gamma \left(I+I_{q}\right).$$

This model is based on the mathematical modelling theories for disease epidemics [10], where, at endemic equilibrium state, we have $\frac{dS}{dt}=\frac{dE}{dt}=\frac{dI}{dt}=\frac{dR}{dt}=0$. Thus, equating *φE* from Equations (3) and (4) and factorizing *I* gives us $\left[\frac{\beta \zeta \left(1-q\right)\left(1-\kappa \right)S}{N}-\left(\varepsilon +\delta +\gamma \right)\right]I=0$. Therefore, assuming initially that the population attains disease-free equilibrium as $\left(S\left(0\right),\;E\left(0\right),\;I\left(0\right),R\left(0\right)\right)=(N,0,\;0,\;0$), then *S/N* = 1. In order for the disease to spread in the population, *I* > 0, resulting in $\beta \zeta \left(1-q\right)\left(1-\kappa \right)-\left(\varepsilon +\delta +\gamma \right)>0.$ Comparing this inequality with the definition of the basic reproduction number, *R_0_* is thus

(8) $$R_{0}=\frac{\beta \left(1-q\right)\left(1-\kappa \right)\zeta }{\delta +\gamma +\epsilon }.$$

### 2.4. Model Simulation

**Figure 2:**
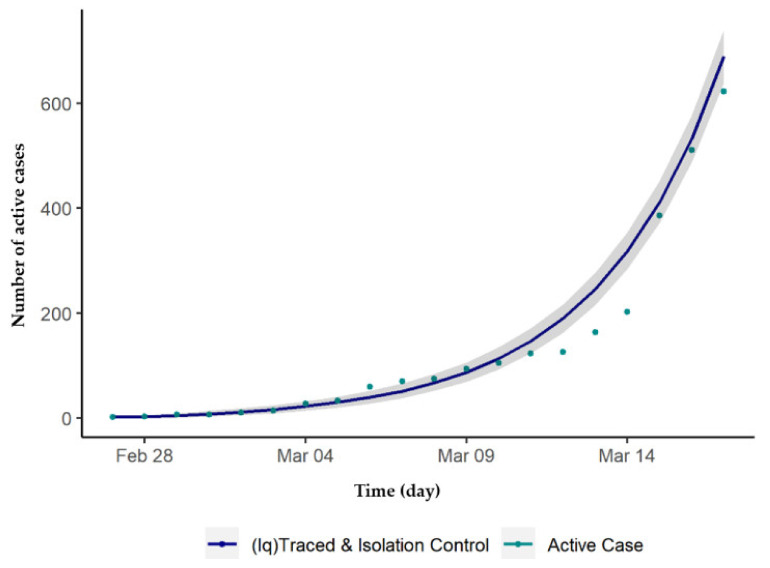
Number of daily active cases and model fit by day, 27 February to 17 March 2020, Malaysia (the grey shades show 95% confidence interval of the fitting).

The transmission model was simulated using R software, Foundation for Statistical Computing, Vienna, Austria [25] with the deSolve package [26]. The model calibration based on the maximum likelihood estimation method utilized the bbmle package, which is written in R language [27]. The ggplot2 package was used to illustrate the simulation in graphs [28]. The parameters chosen to be calibrated were the probability of a susceptible person to become infected per contact (*β*), the average number of contacts per day per case (*ζ*), and the proportion of close contacts traced per day (*q*). Other parameter values were obtained from the MOH and in the literature, as, during the initial stages of the COVID-19 pandemic in Malaysia, data availability was limited. Furthermore, the use of parameters estimated from literature, such as incubation and infectious period, are specific to the COVID-19 pathogen and therefore would have similar pathogenesis across populations; therefore, applying these parameters from existing studies would be appropriate. The reported daily active cases was used to fit the *I_q_* from the model simulations. During the first wave of the outbreak (25 January to 26 February 2020), a total of 22 confirmed cases were reported, of which only 4 were local cases. Due to this, we disregarded this first wave and set 27 February (t0 = 0) as the start of the simulation. Figure 2 shows that the fitting of model (*I_q_*) compared with the total daily active confirmed cases.

**Figure 3:**
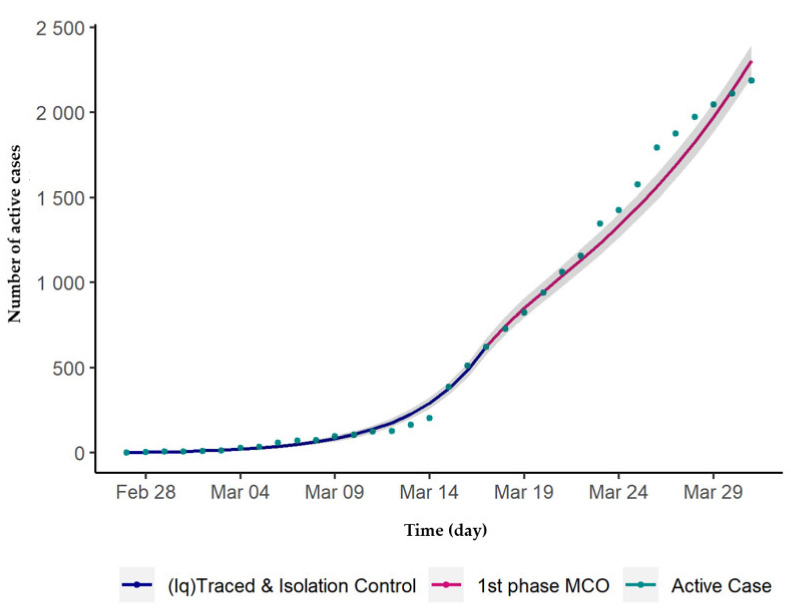
Number of daily active cases and model fit by day, 27 February to 30 March 2020, Malaysia (the grey shades show 95% confidence interval of the fitting).

The calibrated values for the three parameters found were *β* = 0.052, *ζ* = 25, and, *q* = 0.23, determined from the model fit data from 27 February to 17 March 2020, as shown in Figure 2. Malaysia imposed a movement control order (MCO), starting on 18 March 2020, thus, Figure 3 depicts the comparison made between the actual total daily active cases with the model simulation from the 27 February to the first phase of the MCO. For this case, the parameter values were kept as they were, except for *ζ*, which needs to be recalibrated to be 16 as it represents the MCO measures taken. In addition, during this period, there were 43 case fatalities reported and hence *ε* = 0.02.

A plateau was observed during the period from 16–26 February 2020 as there were no new cases reported in Malaysia. However, from 1 March 2020, the number of cases began to increase rapidly with a total number of cumulative COVID-19 cases at 117 on 9 March 2020. In predicting the size of the outbreak, our model requires a longer period of simulation, focusing on the infectious population “I”, as active cases in the community will continue to move freely and potentially spread the virus to the susceptible population if no control measures are instituted.

## 3. Results

This section presents the simulated results of the four phases (pre-MCO, MCO phases 1–3) based on the parameter values estimated in the model.

### 3.1. Outbreak Simulation With No MCO Measures

**Figure 4:**
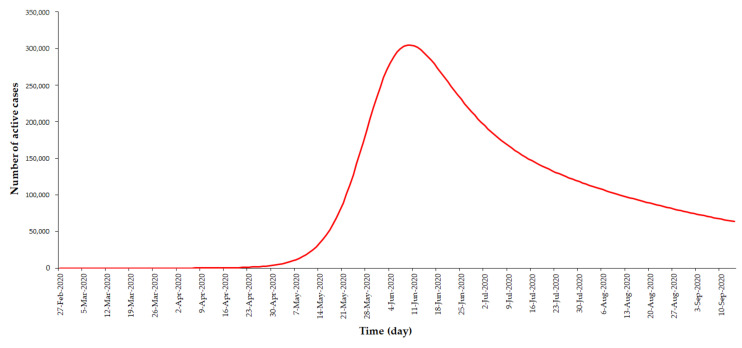
Model simulation of number of COVID-19 active cases by day, with only tracing, isolation, and quarantine measures, but without the MCO, February to September 2020, Malaysia (ζ = 25).

The results of the simulation using this extended SEIR model, but without MCO measures, was represented by the effect of zeta (*ζ*), the value of which was calibrated at 25 based on data from 27 February 2020 to 17 March 2020 (pre-MCO). Based on this simulation, it was estimated that the number of active cases would exponentially rise from May 2020, reaching the peak by mid-June 2020 with a maximum number of 304,907 active COVID-19 cases. In addition, the simulation projected the outbreak to progress for well over 8 months, as shown in Figure 4.

### 3.2. Outbreak Simulation with MCO Measures

**Figure 5:**
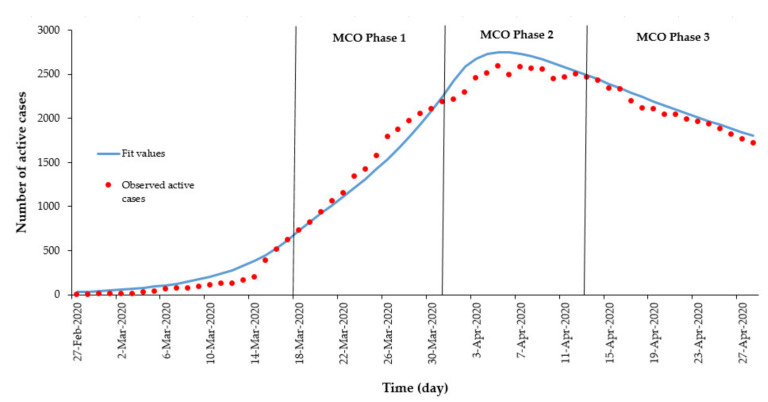
Observed number of COVID-19 active cases and models fit by day at different MCO phases, February to April 2020, Malaysia.

The results of our model fit with observed active cases during the implementation of MCO measures and estimated that the zeta(*ζ*) value showed a downward trend from *ζ* = 25 during the pre-MCO period (before 18 March 2020) to *ζ* = 16 during the first MCO phase and continued to decrease to *ζ* = 7 during the second and third MCO phases. The observed number of active cases rose exponential from March 2020, reaching the peak by early April 2020 with a maximum number of 2596 active COVID-19 cases. In addition, the observed outbreak began to progressively decrease within 3 months from the first cases, as shown in Figure 5.

### 3.3. Comparison of Outbreak Simulation With and Without MCO Measures

During the pre-MCO period, the zeta (*ζ*) value was estimated to be *ζ* = 25, wherein with the implementation of the MCO phase, the *ζ* value progressively decreased from 25–7 (Phase 1 MCO, *ζ* = 16; Phase 2 and 3 MCO, *ζ* = 7). This observation suggests that the number of contacts per case per day significantly reduced with the implementation of the MCO, which in turn decreased the disease transmission, as reflected by the observed reduction in number of active cases.

In addition, the model simulation with no MCO measures showed the estimated peak number of active cases to be 304,907 compared to the observed peak active cases of 2596 during the period where MCO measures were implemented. This was an estimated reduction of 99.1% of peak active cases with the implementation of MCO measures. The peak of the epidemic was observed to occur earlier by 2 months (from June to April 2020) with the implementation of the MCO measures.

Furthermore, the implementation of the MCO measures resulted in the decrease of the epidemic duration, spanning well over 8 months without MCO measures to approximately over 3 months with MCO measures. This would suggest that the flattening of the curve decreased the peak and reduced the duration of the overall epidemic.

## 4. Discussion

Most modelling studies of COVID-19 outbreaks simulate a forecast of cases occurring during an outbreak without taking into account effects of MCO measures. This study extends on a previously developed SEIR model by Labadin and Hong (2020) [29], which now includes traced, exposed, infected, isolated, and quarantined cases. In addition, we utilized data of close contacts, suspected cases (Person Under Investigation), quarantined cases, and isolated cases to develop a more robust model with forecasts of higher accuracy. Furthermore, these forecasts were then used to determine the effectiveness of MCO measures for the COVID-19 outbreak.

The model utilized parameters from existing literature and locally calibrated parameters, namely (i) proportion of close contacts traced per day (*q*), (ii) transmissibility (R_t_), and (iii) probability of a susceptible person becoming infected (*β*) to increase the model representativeness for the Malaysian setting [30]. The proportion of close contacts traced per day (*q*) was 0.23, for which 23% of close contacts were successfully traced in 1 day. As evidence suggests, the ability to trace higher numbers of contacts in a timely manner would increase the chances of detecting cases before they are symptomatic [31], whereas 50% success rate of contact tracing is sufficient to control an outbreak with a minimum R_0_ of 1.5 [32], hence the ability to control the outbreak successfully [32]. Therefore, effective contact tracing measures is crucial in controlling the outbreak by allowing for early case detection, contact tracing, isolation, and quarantine [33].

The estimated R_t_ for the COVID-19 outbreak in Malaysia was 1.68, which suggests that the outbreak was greater than the epidemic threshold and therefore was self-sustaining unless effective control measures were implemented. Existing studies have reported higher R_t_ values that range from 2.50–6.49 [4,6,7,8,34,35]. Our lower estimates may be accounted for local data used during the R_0_ parameter estimation using (*κ*), (*δ*), and (*q*), which is representative of the Malaysian outbreak dynamics. In addition, the first outbreak wave comprised of mostly imported cases, allowing for efficient control measures, which had an impact on reducing the R_t_ values in this study.

Due to the relatively low R_t_ reported in this study, we found that the probability of a susceptible person becoming infected (*β*) following an exposure was reported at 0.05. This suggests that 5% of exposed individuals in Malaysia will ultimately be infected. Previous studies have reported higher probabilities of COVID-19 infection per exposure at 0.1 [36]. Our findings could be affected by the small number of cases reported during the first wave or other exposure-related factors [37]. Despite the low (*β*) value in our study, it is vital that MCO measures be instituted to control the outbreak, as was observed with the exponential rise of cases during the second wave of COVID-19 in Malaysia due to the effect of a mass gathering event.

This study shows that the implementation of MCO measures would effectively reduce the number of contacts per case per day (*ζ*) during the MCO phase as compared to pre-MCO. By reducing *ζ*, the COVID-19 epidemic can be controlled effectively [43,44]. In addition, it was observed that *ζ* continued to decrease with the extension of the MCO into the second and third MCO phases. This finding showed that the MCO was effective in decreasing disease transmission and subsequently suppressing the outbreak [45,46,47]. With these measures in place, the R_t_ would be reduced to less than 1, therefore resulting in the outbreak to lose its ability to sustain [5], which supports our findings in this study. Furthermore, these findings reflect the compliance of the public towards the MCO by practicing social distancing measures.

In addition, the findings from our study showed that MCO measures would reduce the peak number of active COVID-19 cases by 99.1% compared to the pre-MCO model simulation, where no MCO measures were taken. These measures would effectively flatten the epidemic curve by reducing the peak and reducing the outbreak duration. Similar findings on the effectiveness of NPI control measures in reducing the number of COVID-19 cases has been reported in China [38,39] and Singapore [31]. MCO measures would avoid contact and prevent disease transmission, which would eventually break the chain of transmission, therefore ending the outbreak. Furthermore, a lower number of infected individuals would reduce the transmission rates of COVID-19 infection among the susceptible population and lower the mortality rates.

Similar results to our study were reported in China and South Korea, where MCO control measures resulted in the COVID-19 outbreak peaking earlier compared to no control measures taken [40,41,42]. The observed early peaking of the outbreak in Malaysia can also be explained by the occurrence of a mass gathering event in late February 2020, involving more than 16,000 people. This event resulted in the rapid exponential rise of COVID-19 cases, which was observed in March 2020. Despite the rapid exponential rise of COVID-19 cases in Malaysia, the institution of effective of outbreak response activities [41,42], especially the early implementation of the MCO, ensured that the epidemic magnitude was reduced, along with a shorter epidemic duration [43,44].

This study demonstrates the need for quality surveillance data obtained by health authorities, which is crucial for the modelling of infectious diseases. There are several factors that are essential for the quality of data, which include an efficient surveillance system, laboratory diagnostic capacity, and data reporting and management systems [49]. The capacity to make forecasts in this study is a result of the available data quality by the MOH Malaysia, as there is a comprehensive infectious disease surveillance system in Malaysia, which includes the eNotifikasi web-based infectious disease reporting system, which is mandated under The Prevention and Control of Infectious Diseases Act 1988, which provides an effective system that ensures the collection of quality data [50].

## 5. Conclusions

This study recommends the implementation of MCO measures, which are effective in controlling the COVID-19 outbreak in Malaysia, showing an observed reduction of peak active cases by 99.1%. This is evident during the COVID-19 outbreak in Malaysia, whereby the implementation of the MCO measures effectively controlled the outbreak. Our findings suggest that existing isolation and quarantine control measures will only be effective with additional MCO measures implemented. Isolation and quarantine control measures alone are unable to effectively control the outbreak due to issues such as asymptomatic transmission, long transmissibility period, ineffective contact tracing, and isolation practices, and hence MCO measures would effectively address these issues [38,40].

There are several limitations in our model, as COVID-19 is a novel pathogen. This model is an extrapolation of a complex problem and many within the field have taken more complex approaches to the question [4,6,7,8,34]. As such, the forecasts are certainly accompanied by some degree of uncertainty. The limited cases during the Malaysian first wave have also made the estimation of parameters difficult. There was a low approximation of the proportion of close contacts traced per day, which was estimated at 23%. Practically, this estimation is likely to be much higher when the outbreak progresses and may have caused an underestimation of the effect of control measures. Similarly, an average in the number of contacts was used with a large range and a small pool of outliers within the data could have caused an unstable estimate on the number of contacts an infected individual would have. However, with more cases being reported in the second wave, the knowledge exponentiation will certainly improve our understanding of the disease and as such allow more efficient epidemic control. Future studies should aim to include more case counts and spatial dimensions to the model and use time series models to predict COVID-19 outbreaks.

Despite the limitations in the estimation of transmission parameters, the findings of this study support the effectiveness of MCO measures in breaking the chain of COVID-19 transmission dynamics. We suggest that implementation of isolation, quarantine, and especially MCO measures be carried out and sustained until the outbreak stops or until a vaccine becomes available [45].

## Figures and Tables

**Table 1:** Description of the parameters and their corresponding values used in the model simulation.

Parameter	Description	Value	Source
*N*	Total human population in Malaysia	32,600,000	(DOSM 2019)
$\frac{1}{\phi }$	Incubation Period	6.5	[16]
*β*	Probability of susceptible become infectious per contact	0.052	Calibrated using data (27 February to 17 March 2020)
$\frac{1}{\gamma }$	Infectious period	3.6	[4]
*ε*	Death rate due to COVID-19	0	MOH (as per 16 March 2020)
*ζ*	The average number of contacts per day per case	25	Calibrated using data (27 February to 17 March 2020)
*q*	The proportion of close contact traced per day	0.23	Calibrated using data (27 February to 17 March 2020)
$\frac{1}{\theta }$	The duration of quarantine	14	MOH
*κ*	The proportion of exposed persons who performed effective precautions	0.05	[24]
*δ*	The mean daily rate at which infectious cases are isolated	0.03	[24]

Department of statistics Malaysia (DOSM); Ministry of Health Malaysia (MOH).
